# Metacognition and Its Relationship With Orbitofrontal Cortex and Thalamus Volumes in Patients With Obsessive‐Compulsive Disorder

**DOI:** 10.1002/brb3.70716

**Published:** 2025-08-04

**Authors:** Murad Atmaca, Sevler Yildiz, Ismail Taskent, Muhammed Fatih Tabara, Mehmet Gurkan Gurok, Sevda Korkmaz, Osman Mermi, Hanefi Yildirim

**Affiliations:** ^1^ Department of Psychiatry School of Medicine Firat University Elazig Türkiye; ^2^ Clinic of Psychiatry Fethi Sekin City Hospital Elazig Türkiye; ^3^ Department of Radiology Kastamonu University Faculty of Medicine Kastamonu Türkiye; ^4^ Department of Radiology School of Medicine Firat University Elazig Türkiye

**Keywords:** metacognition | obsessive‐compulsive disorder (OCD) | orbitofrontal cortex (OFC) | thalamus

## Abstract

**Purpose:**

The study aims to explore the relationship between orbitofrontal cortex (OFC) and thalamus volumes and metacognition in patients with obsessive‐compulsive disorder (OCD). By analyzing structural MRI data and metacognitive measures, it investigates how brain volume variations correlate with dysfunctional beliefs and OCD symptoms.

**Method:**

The study consisted of 20 patients with OCD and 20 healthy controls. Yale‐Brown Obsession Compulsion Scale (Y‐BOCS), Metacognition Questionnaire‐30 (MCQ‐30), Hamilton Depression Scale (HAM‐D), and Hamilton Anxiety Scale (HAM‐A) were administered to OCD patients and healthy controls. They then underwent structural MRI scans to measure the volume of the OFC and thalamus.

**Finding:**

On both sides, OCD patients had smaller volumes of OFC than healthy control individuals, and their thalamic volumes were similar to those of the control participants. Furthermore, MCQ‐30 scores showed a substantial negative correlation with left OFC volume.

**Conclusion:**

In conclusion, we suggest that dysfunctional metacognitive beliefs might be related to the occurrence of OCD, and these beliefs might be associated with the left side of OFC neuroanatomically.

## Graphical abstract

This study investigates the interplay between metacognitive processes and the neuroanatomical characteristics of the orbitofrontal cortex (OFC) and thalamus in obsessive‐compulsive disorder (OCD). Findings suggest that reduced OFC volumes and specific metacognitive dysfunctions are closely linked to OCD symptoms, highlighting the critical role of these brain regions in understanding and potentially addressing the cognitive mechanisms underlying OCD.

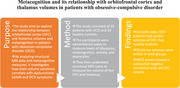

## 1 Introduction

Obsessive‐compulsive disorder (OCD) is a common and disabling disorder that is classified in the Diagnostic and Statistical Manual of Mental Disorders, fifth edition, under obsessive‐compulsive and related disorders (American Psychiatric Association 2013). One of the main features of this disorder is that thoughts become uncontrollable, and people need to engage in compulsive behaviors to cope with them (Huang et al. 2023). OCD affects a significant proportion of the population, making it a significant health concern. Metacognitive theories are important for understanding the pathophysiology of OCD, as the meaning patients attribute to their thoughts and how they cope with them can perpetuate the disorder (Mavrogiorgou et al. 2023). Metacognition is defined as the ability to recognize, control, and restructure one's own thought processes when necessary. In OCD, metacognitive processes involve paying excessive attention to one's thoughts and feeling the need to control them. If OCD patients are unable to control these disturbing thoughts, they may develop the belief that they are real and dangerous. Beliefs about the uncontrollability and danger of thoughts, for example, can increase the severity of obsessions and cause compulsions to recur (Kuhn 2022; McKay et al. 2020). In this context, a pattern for OCD has been proposed that emphasizes how intrusive thoughts can reduce the perceived danger of obsessive thoughts and stimulate metacognitive beliefs about the meaning of thoughts, while also being linked to instrumental beliefs about behavioral responses (Wells and Matthews 1994; Atmaca 2022). Investigating the relationship between metacognitive processes and brain structures could help us to better understand how and why OCD emerges. In particular, identifying the role of critical regions such as the orbitofrontal cortex (OFC) and thalamus in the persistence of obsessive thoughts and repetition of compulsions could inform more effective treatment strategies. Recently, the cortical thickness of various brain areas, including the OFC, has been examined in relation to treatment response prediction in a variety of psychiatric disorders (Szalisznyó and Silverstein 2021).

However, the biological, cognitive, and neurodevelopmental underpinnings of OCD remain poorly understood, which makes it difficult to predict how patients will respond to treatment and how the disorder will progress. A review of functional and structural neuroimaging studies has emphasized the importance of the cortico‐striatal axis, as well as metabolic changes in the caudate nucleus, thalamus, anterior cingulate cortex, and frontal cortex, which are stimulated by the provocation of symptoms (Atmaca et al. 2006, 2007, 2008, 2011; Soriano‐Mas 2021; Atmaca, Mermi, Yildirim et al. 2016; Atmaca, Yildirim, Mermi et al. 2016; Hou et al. 2022; Van Der Straten et al. 2017; Biria et al. 2021; Veltman 2021). These studies have identified some specific regions. In particular, OFC and the thalamus have been emphasized. To further our understanding of OCD, our study team conducted a range of structural and neurochemical investigations in patients with the condition. Consistent with this, we previously examined the volume of key brain regions in patients with OCD, including those with either pure or refractory symptoms. These regions included the OFC, caudate nucleus, thalamus, and anterior cingulate cortex (Atmaca et al. 2006, 2007). In that research, we found that patients with OCD had larger thalamus volumes and significantly lower OFC volumes than healthy individuals. We also found that refractoriness to OCD may be related to both the OFC and thalamus regions. Our study team's examination of the connection between OFC volumes and ego defense styles in OCD patients further supported the hypothesis that the OFC might be a brain region associated with refractoriness. We found that patients’ scores for mature, neurotic, or immature defense styles did not significantly correlate with right OFC volumes. However, there was a substantial correlation between left OFC volumes and ratings of immature defense mechanisms (Atmaca et al. 2011).

Although research to date has revealed certain relationships between metacognitive processes in OCD and structural changes in the brain, an in‐depth examination of these relationships is still lacking. In particular, the link between upper level metacognitive beliefs and volumetric changes in the OFC and thalamus regions of the brain has only been examined in a small number of studies, with no definitive results emerging. Significant gaps remain in our understanding of the effects of metacognitive processes on brain structures and of how these effects interact with the clinical symptoms of OCD. When searching the literature regarding the relationship between the volume of different brain regions and metacognitive measurements, we found nothing. Consequently, given that the OFC and thalamus are two important brain regions in OCD patients, we hypothesized that there might be a relationship between their volumes and metacognition and set out to investigate their interaction. We hypothesize that metacognitive processes such as poor insight and the need to control thoughts in OCD patients will be positively correlated with changes in OFC and thalamus volume.

## 2 Materials and Methods

### 2.1 Subjects and Clinical Evaluation

The study included 20 patients with OCD, as defined by the DSM‐5, and 20 healthy controls. A clinician with experience in the field conducted interviews with individuals who met the criteria for OCD using the Turkish version of the Structured Clinical Interview for DSM‐5 Disorders (SCID‐5) (Elbir et al. 2019; First 2015). The Yale‐Brown Obsessive Compulsive Scale (Y‐BOCS), the Metacognitive Questionnaire‐30 (MCQ‐30), the Hamilton Depression Scale (HAM‐D), and the Hamilton Anxiety Scale (HAM‐A) were administered to patients with OCD and healthy control subjects (Akdemir et al. 1996; Karamustafaoğlu et al. 1993; Tosun and Irak 2008; Yazici et al. 1998; Goodman et al. 1991; Wells and Cartwright‐Hatton 2004; Hamilton 1959, 1960).

Participants were recruited in strict accordance with the terms of the Declaration of Helsinki. Following approval of the study by the Local Ethics Committee at the School of Medicine at Fırat University, written informed consent was obtained from all patients and healthy individuals. The exclusion criteria were as follows: (i) any co‐occurring psychiatric disorder, except depression, whether current or past; (ii) a history of severe head trauma; (iii) any contraindications to an MRI scan, such as a pacemaker or metal implants; (iv) any current medical issues; (v) alcohol or drug abuse within 6 months of the study; and (vi) an unstable dosage of psychoactive medication within 4 weeks of the study. Healthy control subjects were selected from individuals who had applied to the outpatient psychiatry clinic for reasons unrelated to illness (e.g. to obtain a health certificate for employment purposes). The inclusion criteria for healthy controls were as follows: No psychiatric disorder; no medical illness; no history of alcohol or substance abuse in the last six months; and no use of psychotropic drugs in the last four weeks.

### 2.2 Magnetic Resonance Imaging Procedure

**FIGURE 1:**
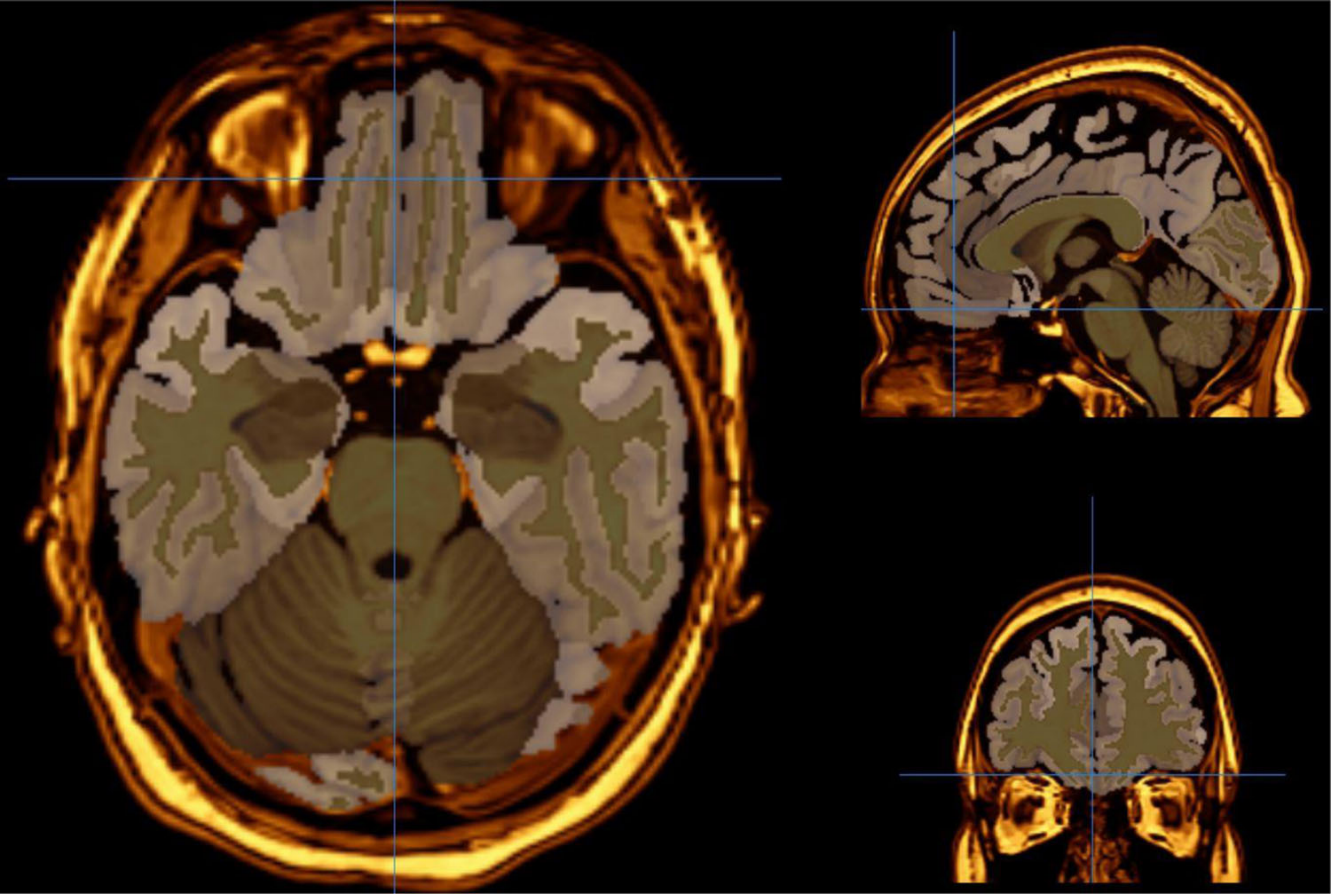
Representative MRI slice depicting the orbitofrontal cortex (OFC). This image illustrates the anatomical localization of the OFC on a T1‐weighted axial MRI slice from a study participant. The region of interest (ROI), segmented using the vol2Brain pipeline and visualized in ITK‐SNAP, is highlighted to demonstrate the boundaries used for volumetric analysis. This region was examined bilaterally to assess structural differences between patients with obsessive‐compulsive disorder (OCD) and healthy controls.

**FIGURE 2:**
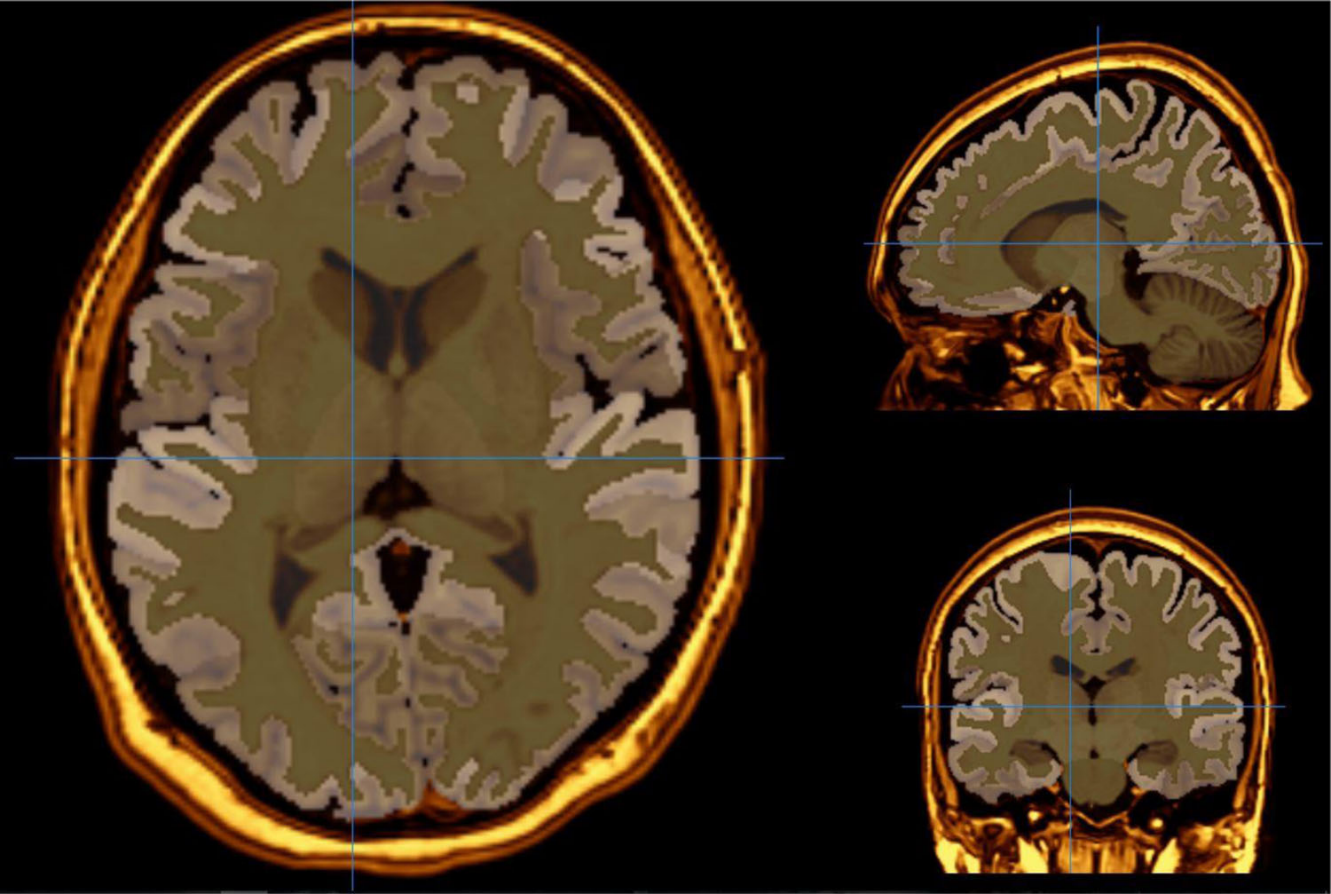
Representative MRI slice depicting the thalamus. This figure shows a T1‐weighted axial MRI slice highlighting the thalamus region, as segmented for volumetric analysis. The thalamus was delineated using the vol2Brain pipeline and rendered in ITK‐SNAP for visual confirmation. Bilateral thalamus volumes were compared between OCD patients and healthy controls to explore associations with metacognitive functioning.

All MRI investigations were performed by using a 3‐T MRI scanner (Signa; GE Medical Systems, Milwaukee, WI, USA). Before the beginning of the procedure, it was told to patients that they could be given an anti‐anxiety drug if required. Imaging parameters were as follows: flip angle = 200, field of view [FOV] = 240 mm, echo time [TE] = 15.6 ms, bandwidth = 20.8, slice thickness = 2.4 mm, echo spacing = 15.6 ms, repetition time [TR] = 2000 ms, and 8 echoes, with a final resolution of 0.9375 mm × 0.9375 mm × 1.328 mm. On the other hand, magnetic field uniformity was tried to provide before every scan. The conversion of 3D T1A data obtained from MRI scans was undertaken from DICOM format to NIfTI format. The data in NIfTI format were uploaded to VolBrain, an automatic brain MRI segmentation program. Volbrain is a free, online, and web‐based data processing system that automatically performs morphometry and voxel‐based volumetry from MRI data. The vol2Brain pipeline facilitates the segmentation of the brain into over 100 segments, with subsequent automated volume measurement. In the present study, the volumes of the relevant regions were measured from T1‐weighted images using the vol2Brain version 1.0 pipeline. The NIfTI format data obtained through vol2Brain were converted into 3D images using the ITK‐Snap application, version 4.0.1. The accuracy of the masking process for the structures whose volumes were investigated was checked by two neuroradiologists (H.Y., who has nearly 30 years of experience in neuroradiology, and I.T., who has 10 years of experience in neuroradiology). Intracranial volume correction was performed. Measurements were repeated bilaterally in 10 selected subjects to determine within‐rater reliability. In this procedure, the intraclass correlation coefficient (ICC) for all regions was found to be above 0.88, which indicates sufficient reliability. Figures 1 and 2 illustrate examples of OFC and thalamus sections, respectively.

### 2.3 Statistical Analysis

All statistical analyses were performed by means of the software the Statistical Package for the Social Sciences, version 16.0 (SPSS Inc., Chicago, IL, USA). Descriptive data for age and other demographic variables, volumetric findings, and scale scores were computed. The continuous variables between OCD patients and healthy control participants were compared using an independent sample *t*‐test, and the chi‐square test was employed for categorical data. As the HAM‐D and HAM‐A scores of the OCD group and the healthy controls were found to be significantly different, these variables were evaluated in the general linear model, and the volumes were compared separately. We also used the Pearson correlation coefficient to evaluate correlational relationships between volumetric data and clinical and demographic variables or scale scores. To control for the increased risk of type I errors resulting from multiple comparisons, the Benjamini–Hochberg false discovery rate (FDR) correction was applied to the correlation analyses and volumetric group comparisons. This method was chosen because it provides a balance between limiting false positives and maintaining statistical power, which is particularly important in studies with modest sample sizes, like this one. A value of 0.05 was set as the significance threshold.

## 3 Results

**FIGURE 3:**
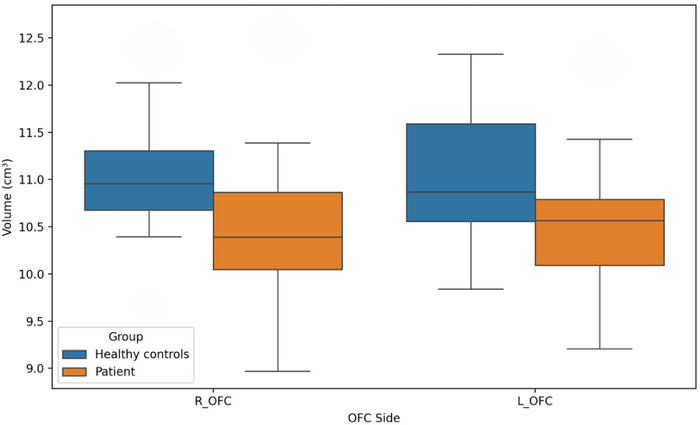
Boxplots of orbitofrontal cortex (OFC) volumes in obsessive‐compulsive disorder (OCD) patients and healthy controls. Boxplots comparing the left and right OFC volumes (in cm^3^) between patients with OCD and healthy control participants. Central lines indicate medians; box edges represent interquartile ranges (IQR). OCD patients showed significantly lower bilateral OFC volumes.

**FIGURE 4:**
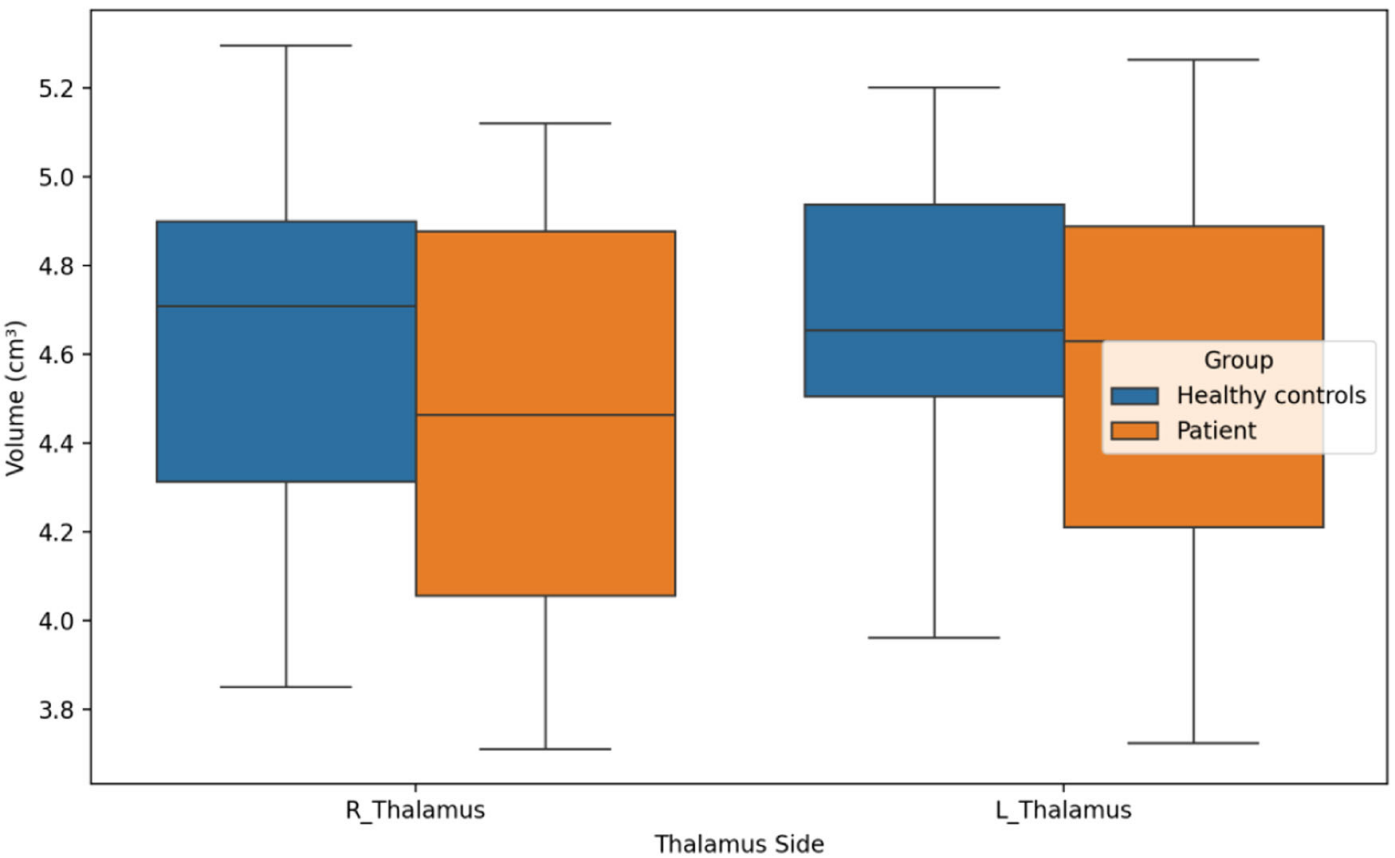
Boxplots of thalamus volumes in OCD patients and healthy controls. Boxplots comparing the left and right thalamus volumes (in cm^3^) between patients with OCD and healthy control participants. Central lines indicate medians; box edges represent interquartile ranges (IQR). No statistically significant difference was found in the thalamus volumes of the groups.

There were significant differences in the mean ages of the groups (29.15 ± 8.04 vs. 23.85 ± 2.48, *p* = 0.028). There were no significant differences in sex distribution, handedness, or other sociodemographic variables. None of the participants had a history of drug or alcohol abuse. Regarding the scale scores, there was a significant difference in Y‐BOCS scores between patients with OCD and healthy control subjects (respectively, 21.25 ± 6.36 vs. 6.18 ± 3.32, *p* < 0.001). In addition, we found remarkable differences in the HAM‐D and HAM‐A scores. In the patient group, the mean HAM‐D score was 30.85 ± 8.25 versus 5.53 ± 2.81 for healthy comparison subjects, and the mean HAM‐A score was 23.00 ± 9.83 versus 5.46 ± 4.72, with a *p* level of <0.001 for both. We found a statistically significant difference in MCQ‐30 scores between patients with OCD and healthy controls (mean MCQ‐30 score of 76.20 ± 12.79 for patients vs. 55.92 ± 13.19 for controls, *p* < 0.001). We found that patients with OCD had smaller OFC volumes than healthy control subjects on both sides. The mean volumes of the left OFC were 10.49 ± 0.70 and 11.01 ± 0.69 cm^3^, respectively, for patients with OCD and healthy control subjects (*p* = 0.02). Similarly, the mean volumes of the right OFC were 10.50 ± 0.80 and 11.02 ± 0.72 cm^3^, respectively, for patients with OCD and healthy controls (*p* = 0.03). In the analyses in which age, HAM‐D, and HAM‐A were taken as confounding factors, it was observed that the significant difference in OFC volumes between the groups persisted (*F* value for right OFC = 0.665; *F* value for left OFC = 0.643). Box plot graphs for the comparison of OFC volumes of the groups are shown in Figure 3. Subsequent to thorough analysis of thalamus volumes, the thalamus volume of the patient group was found to be decreased in comparison with the control group, though this was not found to be statistically significant. Box plot graphs for the comparison of thalamus volumes of the groups are shown in Figure 4. The sociodemographic data, scale scores, and volumetric comparisons of the groups are presented in Table 1.

**TABLE 1:** Clinical, demographic, and volumetric parameters of patients and healthy subjects.

	Patient group (*n* = 20)	Healthy controls (*n* = 20)	*P*
Sex (female/male)	11/9	11/9	1.000^a^
Age	29.15 ± 8.04	23.85 ± 2.48	**0.028** ^b^
Education High school and above Primary school and below			0.49^a^
	15	13	
	5	7	
Marital status (single/married)	13/7	12/8	0.744^a^
Handedness (right)	20	20	1.000^a^
Y‐BOCS	21.25 ± 6.36	6.18 ± 3.32	**< 0.001** ^b^
HAM‐D	30.85 ± 8.25	5.53 ± 2.81	**< 0.001** ^b^
HAM‐A	23.00 ± 9.83	5.46 ± 4.72	**< 0.001** ^b^
MCQ‐30	76.20 ± 12.79	55.92 ± 13.19	**< 0.001** ^b^
Orbitofrontal cortex Left Right	10.49 ± 0.70 10.50 ± 0.80	11.01 ± 0.69 11.02 ± 0.72	**0.02** ^b^ **0.03** ^b^
Thalamus Left Right	4.52 ± 0.42 4.45 ± 0.48	4.66 ± 0.39 4.61 ± 0.40	0.33^b^ 0.31^b^

*Note*: Volumes presented are in cm^3^.

Abbreviations: HAM‐A, Hamilton Anxiety Scale; HAM‐D, Hamilton Depression Scale; MCQ‐30, Metacognition Questionnaire‐30; Y‐BOCS, Yale‐Brown Obsession Compulsion Scale.

^a^ Chi‐square test.

^b^ Student's *t*‐test.

To detect the relationship between region volumes and scale scores, we used Pearson's correlation test. We found that there was not any correlation between the Y‐BOCS scores and OFC volumes in both sides (for the left OFC, *p* = 0.16; for the right OFC, *p* = 0.16). On the other hand, we detected that there was not any correlation between the Y‐BOCS scores and thalamus volumes in both sides (for the left thalamus, *p* = 0.56; for the right thalamus, *p* = 0.73). Furthermore, MCQ‐30 scores showed a substantial negative correlation with left OFC (*r* = −0.39, *p* = 0.025). We did not find any other correlational relationships between OFC and thalamus volumes and any scale scores and clinical and demographic variables.

## 4 Discussion

The results of the study demonstrated that patients diagnosed with OCD exhibited reduced volumes of OFC on both sides when compared to the healthy control participants. Additionally, the volumes of thalamus on both sides were found to be similar to those observed in the healthy control participants. Furthermore, a significant correlation was observed between MCQ‐30 scores and left OFC. In consideration of the aforementioned findings, it can be posited that a bidirectional relationship may exist between metacognitive processes (i.e., the interpretation and management of thoughts) and abnormalities in critical brain structures, such as the OFC and thalamus, in individuals diagnosed with OCD. The initial component of these results is consistent with the earlier finding that patients diagnosed with OCD exhibited reduced volumes of the OFC in comparison to healthy controls (Atmaca et al. 2006, 2007). The final section of the results on MCQ‐30 scores is evidently novel. As was discussed in introduction, metacognition is defined as the cognitive processes and structures that monitor and regulate an individual's own cognition. In this context, a model of OCD has been advanced, emphasizing the manner in which intrusive thoughts can both diminish the perceived risk associated with obsessive thoughts and stimulate metacognitive beliefs, which are linked to the meaning of thought. These metacognitive beliefs in turn act as a catalyst for instrumental beliefs connected to behavioral responses (Wells and Matthews 1994). MCQ‐30 has been shown to have a high degree of accuracy in the detection of metacognitions that are particularly unhelpful. These metacognitions have been found to be associated with the occurrence of obsessive and compulsive symptoms, pathological worry, and trait anxiety. In this particular context, it is important to note that our findings, which demonstrate a negative correlation between MCQ‐30 scores and left OFC volumes, seem to be of significance. First, this significant correlation suggests the possibility of a relationship between left OFC and the presence of obsessive and compulsive symptoms, pathological worry, and trait anxiety. Obsessive thoughts are believed to be negatively attributed to metacognitive beliefs concerning the perceived importance and/or the potentially harmful consequences of thinking a particular thought. These beliefs are thought to aim to regulate the self through self‐beliefs and influence the meanings and functions of cognition (Wells and Matthews 1994). In this way, Taylor et al. (2006) reported that some OCD patients exhibited overt dysfunctional beliefs associated with obsessive‐compulsive symptoms, whereas others did not, thereby suggesting that dysfunctional beliefs may only contribute to a subset of OCD cases. In conjunction with this finding, it can be hypothesized that dysfunctional metacognitive beliefs may be associated with left OFC. The thalamus, anterior cingulate cortex, caudate nucleus, and OFC are the key brain regions associated with OCD. In a previous study, the volumes of these regions in patients with either pure or refractory symptoms were examined, and it was found that OCD patients had statistically significantly greater thalamus volumes and smaller OFC volumes than healthy controls. This finding indicates a potential correlation between refractoriness to OCD and these specific regions (Atmaca et al. 2006, 2007). The results of this study align with the findings of the aforementioned research. Conversely, our research team previously examined the relationship between ego defense styles and OFC volumes in OCD patients. The findings revealed that the right side of the OFC was not associated with the utilization of immature defense styles in OCD, whereas the left side was linked to refractoriness to OCD (Atmaca et al. 2011). Our previous finding appears to be in accordance with our result here, which demonstrates an association between left OFC and metacognition. In the meantime, recent studies have measured the cortical thickness of various brain areas, including the OFC, in a range of psychiatric disorders, especially in regard to the treatment response prediction (Hoexter et al. 2015). Hoexter et al. investigated the potential of OFC thickness as a biomarker to differentiate OCD outcomes. The study revealed that the thicknesses of the left and right medial OFC were the most discriminatory measures. This finding suggests that OFC thickness is a strong predictor of treatment response in OCD patients who have not yet received treatment (Hoexter et al. 2015).

The left OFC is closely associated with speech and reasoning, whereas the right OFC is strongly linked to emotional experiences (Kang et al. 2015). This study's finding that dysfunctional metacognitive beliefs are associated with the left OFC supports this claim. Contrary to our study, there are also studies that found the right OFC to be more associated with OCD (Bowen et al. 2021). This discrepancy underscores the intricate nature of the OFC's function in diverse psychological conditions. Further research is required to elucidate the specific functions of the left and right OFC in the context of OCD and how they may interact with metacognitive beliefs.

Prior to concluding the discussion section, it is imperative to articulate the study's limitations. A primary limitation of the study is the relatively small sample size, which restricts the generalizability of the findings and reduces the statistical power to detect subtle associations between brain volumes and metacognitive measures. The cross‐sectional design of the study also limits causal interpretations. It remains unclear whether structural brain differences contribute to metacognitive dysfunction or vice versa. Furthermore, although the total MCQ‐30 scores were examined, no investigation was made into the relationship between the subscales of the MCQ‐30 and the volumes of the OFC and thalamus. This limitation impedes our capacity to discern metacognitive domains with greater specificity, which may possess neuroanatomical relevance. Furthermore, despite endeavors to regulate for confounding factors such as depression and anxiety, residual confounding cannot be entirely discounted. The use of volumetric MRI data in isolation, devoid of functional imaging or longitudinal follow‐up, further restricts the attainment of a more comprehensive understanding of the dynamic interplay between brain structure and cognitive processes in OCD. Finally, the study did not explore the potential effects of medication history or symptom subtypes, which might influence both brain morphology and metacognitive profiles.

## 5 Conclusion

OCD is a complex disorder in which mental processes and brain structures are intertwined. Research suggests that the metacognitive impairments in OCD (e.g., the belief that thoughts are uncontrollable and dangerous, over‐attribution of importance to obsessions, lack of insight, and habits such as thought suppression) are closely related to abnormalities in the OFC‐thalamus circuitry in the brain. The reduced volume of the OFC and changes in the volume of the thalamus in OCD patients may be a reflection of their internal relationship with the world (the way they perceive and manage their thoughts). The overworking of the orbitofronto‐thalamic circuit in these patients manifests as catastrophizing every mistake and perceiving every thought as real, whereas misinterpretations at the metacognitive level also lead to the stimulation of this brain circuit. In conclusion, we propose that dysfunctional metacognitive beliefs may be linked to the development of OCD and that these beliefs may be associated with the left side of the OFC neuroanatomically. Because of the small sample size in the current study, new research is needed to give additional support for the findings.

## Author Contributions

**Murad Atmaca**: conceptualization, project administration, writing – original draft, supervision. **Sevler Yıldız**: writing – original draft, data curation, investigation, funding acquisition. **Ismail Taskent**: software, resources, validation, writing – review and editing. **Muhammed Fatih Tabara**: investigation, writing – review and editing, data curation. **Mehmet Gurkan Gurok**: writing – review and editing, formal analysis, methodology. **Sevda Korkmaz**: investigation, data curation, supervision, writing – review and editing. **Osman Mermi**: investigation, data curation, writing – review and editing, supervision. **Hanefi Yildirim**: software, supervision, resources, validation, visualization, writing – review and editing.

## Peer Review

The peer review history for this article is available at https://publons.com/publon/10.1002/brb3.70716

## Data Availability

The data that support the findings of this study are available on request from the corresponding author. The data are not publicly available due to privacy or ethical restrictions.
